# Cyclization Reaction of Acyl Thiourea Chitosan: Enhanced Antifungal Properties via Structural Optimization

**DOI:** 10.3390/molecules23030594

**Published:** 2018-03-06

**Authors:** Yukun Qin, Weixiang Liu, Ronge Xing, Song Liu, Kecheng Li, Pengcheng Li

**Affiliations:** 1 Key Laboratory of Experimental Marine Biology, Institute of Oceanology, Chinese Academy of Sciences, No. 7 Nanhai Road, Qingdao 266071, China; lwx_1009@126.com (W.L.); xingronge@qdio.ac.cn (R.X.); sliu@qdio.ac.cn (S.L.); lkc@qdio.ac.cn (K.L.); 2 Laboratory for Marine Drugs and Bioproducts of Qingdao National Laboratory for Marine Science and Technology, No. 1 Wenhai Road, Qingdao 266237, China; 3 University of Chinese Academy of Sciences, Beijing 100049, China

**Keywords:** synthesis, 1,2,4-triazolyl, acyl thiourea, chitosan, antifungal activity

## Abstract

In this study, 3-methyl-1,2,4-triazolyl chitosan (MTACS) and 3-chloromethyl-1,2,4-triazolyl chitosan (CMTACS) were prepared via cyclization of acyl thiourea chitosan (TUCS). Their structures were confirmed by FT-IR, ^1^H-NMR, elemental analysis, DSC, XRD, and SEM. The conformations were predicted using the Gaussian 09 program. Additionally, the antifungal properties of MTACS and CMTACS against *Stemphylium solani* weber (*S. solani*), *Alternaria porri* (*A. porri*), and *Gloeosporium theae-sinensis* (*G. theae-sinensis*) were assayed in vitro and ranged from 250 μg/mL to 1000 μg/mL. The results showed that MTACS and CMTACS exhibited enhanced inhibitory effect on the selected fungi compared to the original chitosan and TUCS. In particular, they displayed better antifungal activities against *A. porri* and *G. theae-sinensis* than that of the positive control, Triadimefon. The findings described here may lead to them being used as antifungal agents for crop protection.

## 1. Introduction

Plant-pathogenic fungi can lead to a decrease in crop yield and quality, resulting in huge economic losses every year worldwide [1]. For example, onion purple blotch disease, which is caused by *A. porri*, can bring about serious yield losses through loss of leaf tissue and a subsequent reduction in the rate of bulb development [2]. *S. solany*, the causal pathogen for grey leaf spot in tomatoes and leaf blight in alliums and cotton, can cause significant damage [3]. It has also been shown to be a significant cause of disease in Chinese garlic crops [4]. Anthracnose caused by *G. theae-sinensis* is one of the most severe diseases that can affect the growth of leaves and cause serious yield losses of tea [5]. For a long time, synthetic fungicides have been used as the main approach to controlling plant-pathogenic fungi. However, the increasing resistance and potential threats to non-target organisms and environment associated with chemical fungicides have gained more and more attention [6,7]. The safety of pesticides has become a focus of global concern [8,9]. Hence, it is critical to find and develop new environmentally benign pesticides.

Exploring new fungicide candidates from natural compounds has attracted much attention [10,11]. Chitosan (CS), one kind of natural polysaccharide mainly derived from the shells of shrimp and crabs, possesses good biocompatibility, biodegradability, and non-toxicity [12,13]. It has been proven that chitosan has broad-spectrum antifungal activity [14,15], and so it has received much attention in the field of agriculture. However, the application of chitosan in crop protection has been restricted due to its relatively low antifungal activity. Hence, many strategies were proposed to overcome these limitations. A widely used approach is the chemical modification of chitosan [16]. The present amino and hydroxyl groups of chitosan provide scope for structural modifications to improve its solubility and extend its application. Lots of researchers aimed to enhance the antifungal activity of chitosan via introduction of a bioactive group. For example, quaternarization can improve both the solubility and the antimicrobial activity of chitosan [17,18]. Binding with alkyl and acyl groups can also enhance the antifungal properties of chitosan [19,20,21]. Many studies demonstrated that chitosan bearing some antifungal groups such as 1,2,3-triazole [22], thiourea [23], thiosemicarbazones [24], or α-aminophosphonates [25] exhibited enhanced antifungal properties.

Thiourea chitosan (TUCS) derivatives are a kind of chitosan derivative in which C2 amino groups or C6 hydroxyl group are substituted by thiourea groups [26]. They are well documented for their adsorption capacity for heavy metals, and antibacterial, and antifungal activities [27]. Recently, the antifungal properties of thiourea chitosan derivatives have attracted considerable interest. Several papers have reported that TUCS are better fungicidal agents than pure CS [28,29]. In a previous study, we also described the synthesis of acyl thiourea derivatives of chitosan and their antifungal activities. We showed that the acyl thiourea chitosan derivatives had enhanced inhibitory effects compared with the original chitosan [30]. Interestingly, it is known that thiourea is a key intermediate for the synthesis of 1,2,4-triazoles [31]. 1*H*-1,2,4-triazole compounds are among the most important fungicides and play a great role in crop protection [32]. Therefore, it is inferred that structural optimization of TUCS may enhance its activity. To the best of our knowledge, there have been no reports on the preparation and antifungal activity of 1,2,4-triazoles chitosan derivatives via the cyclization of acyl thiourea chitosan (TUCS).

**Scheme 1:**
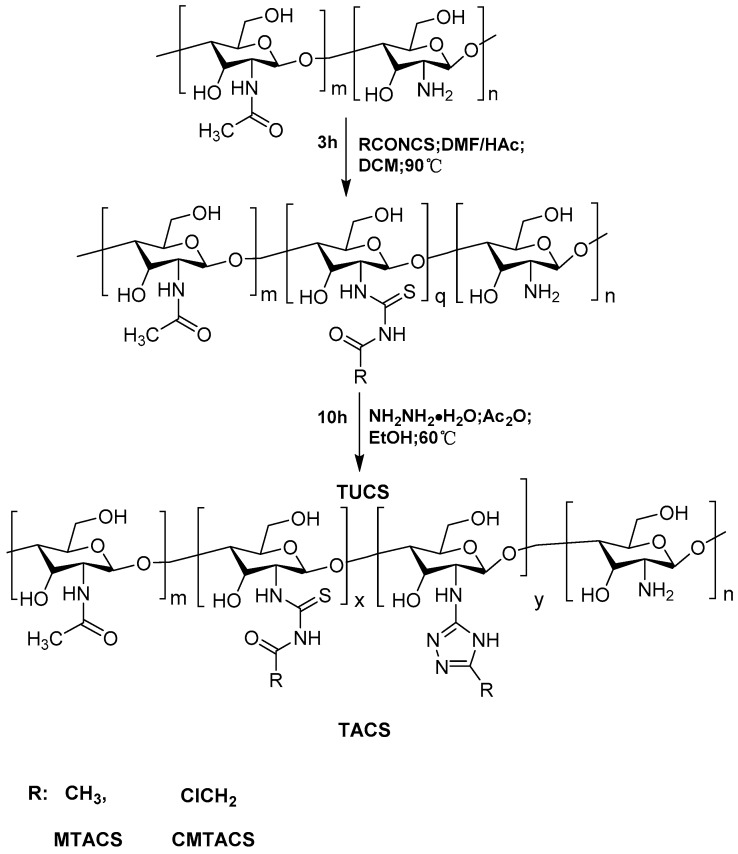
Synthetic route of 3-methyl-1,2,4-triazolyl chitosan (MTACS) and 3-chloromethyl-1,2,4-triazolyl chitosan (CMTACS).

Hereby, with an aim to develop chitosan derivatives with acceptable properties for potential applications in crop protection, 3-methyl-1,2,4-triazolyl chitosan (MTACS) and 3-chloromethyl-1,2,4-triazolyl chitosan (CMTACS) (Scheme 1) was prepared via cyclization of acyl thiourea chitosan (TUCS). Moreover, their antifungal properties were also investigated in vitro.

## 2. Results and Discussion

### 2.1. Preparation and Characterization of 3-Methyl-1,2,4-triazolyl Chitosan (MTACS) and 3-Chloromethyl-1,2,4-triazolyl Chitosan (CMTACS) Derivatives

Ring closure of acyl thiourea in the alkaline medium is a well-known method for the synthesis of 1,2,4-triazoles. Hence, the acyl thiourea chitosan derivatives (TUCS) we reported before were expected to be easily cyclized into corresponding 1,2,4-triazolyl chitosan (TACS) derivatives. The synthesis of 3-methyl-1,2,4-triazolyl chitosan (MTACS) and 3-chloromethyl-1,2,4-triazolyl chitosan (CMTACS) derivatives was accomplished in the current study according to the reaction scheme outlined (Scheme 1). First, acyl thiourea chitosan (TUCS) was obtained by the reaction of acyl isothiocyanates with chitosan. Then acyl thiourea chitosan (TUCS), by refluxing with hydrazine hydrate under a catalyzed amount of acetic anhydride, were cyclized into corresponding 1,2,4-triazolyl chitosan (TACS) derivatives. This reaction may also be extended to other acyl thiourea chitosan derivatives. However, the expected 3-phenyl-1,2,4-triazolyl chitosan (PhTACS) product was not formed, maybe because of the steric hindrance effect of phenyl in benzoyl thiourea chitosan. For this reason, we only describe here the synthesis and antifungal properties of 3-methyl-1,2,4-triazolyl chitosan (MTACS) and 3-chloromethyl-1,2,4-triazolyl chitosan (CMTACS) derivatives. The targeted chitosan derivatives were characterized by FT-IR, ^1^H-NMR, elemental analysis, XRD, SEM, and DSC.

**Figure 1:**
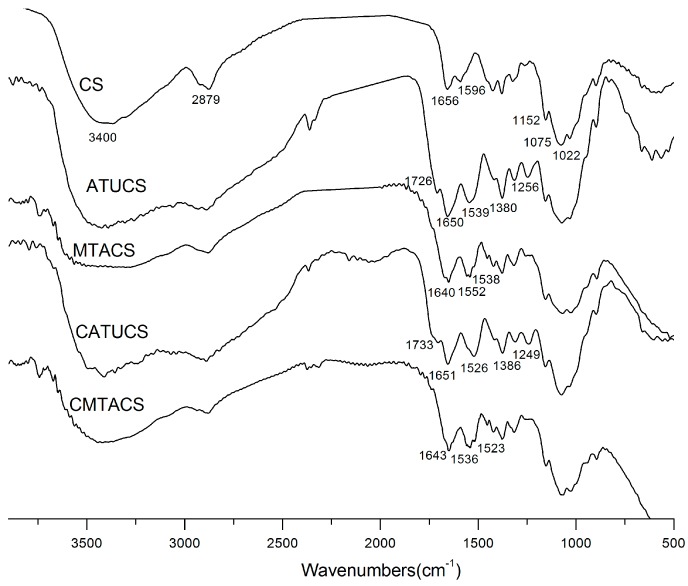
FT-IR spectra of chitosan (CS), acetyl thiourea chitosan (ATUCS), 3-methyl-1,2,4-triazolyl chitosan (MTACS), chloracetyl thiourea chitosan(CATUCS) and 3-chloromethyl-1,2,4-triazolyl chitosan (CMTACS) derivative.

The FTIR spectra of chitosan, acetyl thiourea chitosan (ATUCS), 3-methyl-1,2,4-triazolyl chitosan (MTACS), chloroacetyl thiourea chitosan (CATUCS), and d 3-chloromethyl-1,2,4-triazolyl chitosan (CMTACS) are presented in Figure 1. The broad peak around 3400 cm^−1^ can be assigned to the stretching vibration of OH and the vibration of NH. For ATUCS, there were two new peaks that can be attributed to the C=O and C=S groups appearing at 1726 cm^−1^ and 1256 cm^−1^, respectively. Meanwhile, the intensity of the peak at 1596 m^−1^ (NH_2_) decreased, which indicated that the free amino group of chitosan reacted with acetyl isothiocyanates. These results were in accordance with an earlier study [30]. Compared with ATUCS, for MTACS, the peaks at 1726 cm^−1^ and 1256 cm^−1^ became weaker. Furthermore, a strong new peak was observed at 1640 cm^−1^, assigned to the characteristic absorbance of triazolyl (C=N) group, and confirming the formation of triazole chitosan derivative (MTACS). Similarly, new peaks at 1733 cm^−1^ and 1249 cm^−1^ in the spectra of CATUCS were characteristic of the absorbance of C=O and C=S. A new peak appeared at 1643 cm^−1^ (C=N) in comparison with CATUCS, which indicated that CMTACS was formed.

**Figure 2:**
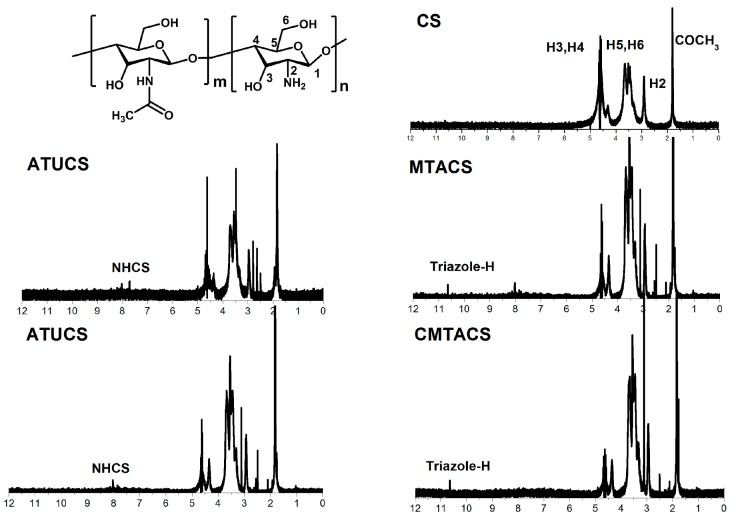
^1^H-NMR spectra of chitosan (CS), acetyl thiourea chitosan (ATUCS), 3-methyl-1,2,4-triazolyl chitosan (MTACS), chloracetyl thiourea chitosan (CATUCS), and 3-chloromethyl-1,2,4-triazolyl chitosan (CMTACS) derivative.

The structure of MTACS was further confirmed by ^1^H-NMR. As shown in Figure 2, in the ^1^H-NMR spectra, all protons were seen accordingly to the expected chemical shift and integral values and the signals of CS, ATUCS, CATUCS, MTACS, and CMTACS were well separated. The peaks at 1.82, 2.93, 3.46–3.54, and 3.62–3.72 ppm were attributed to acetyl-H, H2, (H5 and H6), and (H3 and H4) of the chitosan backbone, respectively. Except for these peaks of chitosan, for MTACS, the signal around 7.70 ppm (NHCS) became weak compared with ATUCS, and a new signal was observed at 10.65 ppm, which was attributed to the triazolyl (NH) group. Meanwhile, for CMTACS, the disappearance of NHCS (CATUCS) at around 8.02 ppm and the new peak found at 10.67 ppm were evidence of ring closure. All of the spectra results indicated the successful synthesis of MTACS and CMTACS.

The degrees of substitution (DS) of ATUCS, CATUCS, MTACS, and CMTACS were calculated on the basis of the percentage. As illustrated in Table 1, the DS_thiourea_ of ATUCS and CATUTS was 33.7% and 27.0%, respectively. Obviously, the DS is not high, which may be the cause of the heterogeneous reaction. For TACS, the DS_triazole_ of MTACS and CMTACS was 22.4% and 19.0%, respectively. It can be inferred that only parts of acyl thiourea group cyclized into corresponding triazolyl group. This was also consistent with the above results.

**Figure 3:**
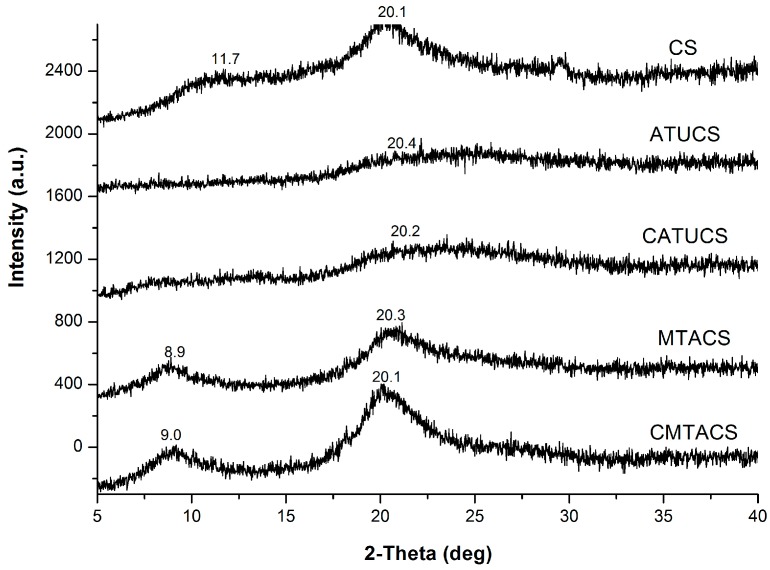
XRD patterns of chitosan (CS), acetyl thiourea chitosan (ATUCS), 3-methyl-1,2,4-triazolyl chitosan (MTACS), chloracetyl thiourea chitosan (CATUCS), and 3-chloromethyl-1,2,4-triazolyl chitosan (CMTACS) derivative.

X-ray diffraction can provide information about the structure of polysaccharide chains as well as their spatial order [33]. The XRD patterns of CS, ATUCS, CATUCS, MTACS, and CMTACS are shown in Figure 3. The characteristic peaks of chitosan was observed at 2θ = 11.7°, 20.1°. The reflection at 2θ = 11.7° attributed to crystal forms I. The strongest peak at 2θ = 20.1° was assigned to crystal forms II. For ATUCS, it was found that the peak exhibited at 2θ = 11.7° disappeared and the peak at 20.1° became weak. This may be caused by the introduction of the acyl thiourea group to the chitosan chain, which causes the hydrogen bond to be destroyed. It indicated that the crystalline structure of chitosan changed slightly through chemical modification. Meanwhile, it also suggested that the original crystallinity of chitosan was not fully destroyed. This also coincided with the above results. In comparison with chitosan and ATUCS, it was obvious that the crystallization of the MTACS derivative was enhanced after the ring closure reaction. A new reflection appeared at 8.9°, together with a strong peak observed at 20.3°. Similarly, for ATUCS, we observed the disappearance of the peak at 2θ = 11.7° and a weak peak at 20.1°. Compared with CS and CATUCS, there were two strong peaks found at 2θ = 9.0° and 20.1°. This shows that the newly formed chitosan derivative changed from the original amorphous structure to a relatively ordered crystalline structure [34].

**Figure 4:**
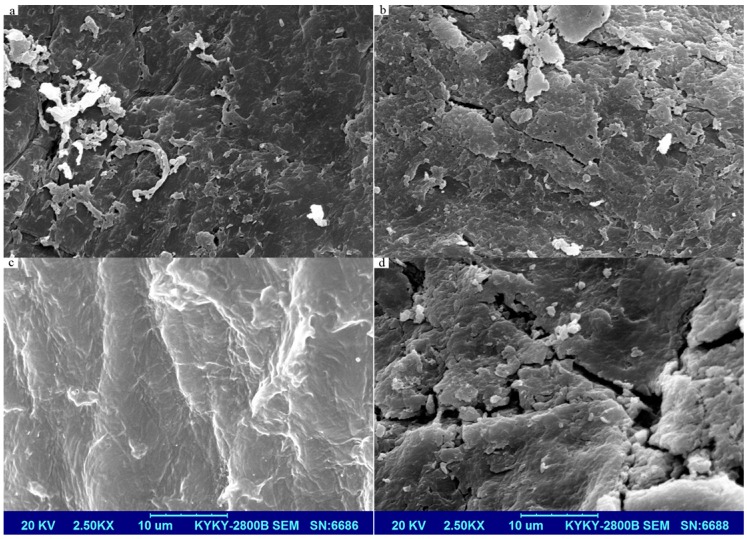
Surface morphology of ATUCS (**a**); CATUCS (**b**); MTACS (**c**); and CMTACS (**d**).

As shown in Figure 4, ATUCS and CATUCS exhibited a compact and relatively smooth structure, while MTACS and CMTACS had a porous structure. It was inferred that the improved solubility of MTACS and CMTACS was related to its more orderly and loose structure. This was also in accordance with the XRD results.

**Figure 5:**
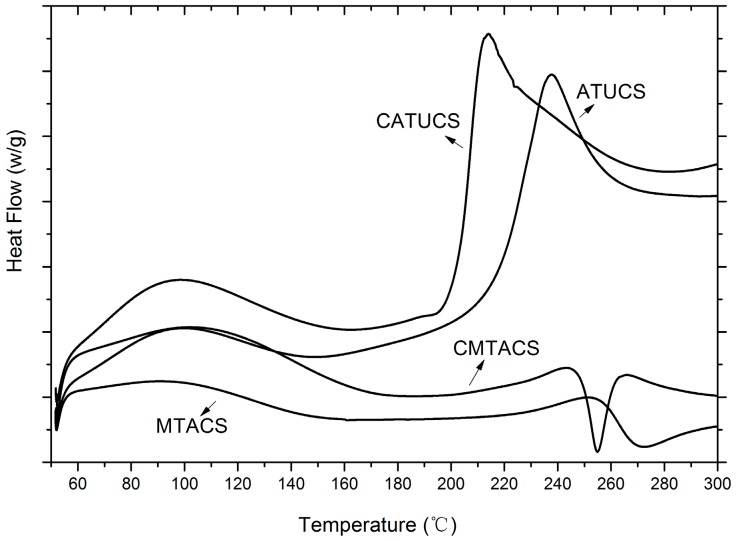
DSC curves of acetyl thiourea chitosan (ATUCS), 3-methyl-1,2,4-triazolyl chitosan (MTACS), chloracetyl thiourea chitosan (CATUCS), and 3-chloromethyl-1,2,4-triazolyl chitosan (CMTACS) derivative.

Moisture influences both the crystalline and amorphous phases of polysaccharides. Therefore, the endothermic peak was expected to reflect physical and molecular changes during chemical modification [35]. DSC has been widely used for determination of thermal stability of polysaccharides, which can reflect physical and molecular changes via structural modification. Figure 5 presents the DSC curves of ATUCS, CATUCS, MTACS, and CMTACS. It can be seen that all the chitosan derivatives have a broad endothermic peak ranging from 90 to 140 °C, which is due to the loss of water or other small molecules. For ATUCS, There was a sharp exothermic peak around 240 °C, which may be owing to the decomposition of the chitosan skeleton. Similarly, CATUCS exhibited a sharp exothermic peak at about 210 °C. Compared with TUCS, it was observed that two sharp peaks appeared at around 270 °C and 255 °C. Therefore, it was inferred that, after the cyclization reaction, the thermal decomposition temperature of the newly formed chitosan derivatives increased and the thermodynamic stability enhanced. This was consistent with the results of XRD and SEM. The crystallinity of MTACS and CMTACS was obviously higher than that of ATUCS and CATUCS. The molecular structure is more ordered, so the thermodynamic properties are more stable.

### 2.2. Antifungal Activity of 3-Methyl-1,2,4-triazolyl Chitosan (MTACS) Derivative

The current research on computational chemistry and structure–activity relationships has played an important role in the development of new pesticides [36]. For example, molecular geometry optimizations can recognize the behavior of a multi-atom system because it provides key information for the prediction of chemical, physical, and biological phenomena of a compound. In this study, we applied the DFT approach to optimize the structure of TUCS and TACS. For TUCS, it was found that the groups grafted on chitosan form a super-conjugated system. Nitrogen atoms, carbon atoms, oxygen atoms, and sulfur atoms are all on the same plane, forming a nearly six-membered ring configuration. Electronegativity analysis showed that the electron cloud density of N-C-N structure in the molecule was low, and shifted to C=O as a whole, which reduced the ability of nitrogen atoms to combine with hydrogen ions to form cations. The sulfur atom in the C=S group approximates neutrality and does not have the ability to bind to hydrogen ions. For TACS, the triazolyl group grafted onto chitosan formed a hyper-conjugation effect with the nitrogen atoms on the chitosan unit, which belonged to a plane. Two nitrogen atoms in the triazolyl group showed strong electronegativity and had a strong ability to bind hydrogen ions. Hence, it was inferred that the different ability to bind hydrogen ions may lead to different antifungal properties. The strong negative charge of TACS may help to improve its antifungal activities.

It was proved that chitosan and its derivatives have broad-spectrum antifungal activity against a variety of fungi [37]. To understand the inhibitory effects of TACS on the selected crop pathogen fungi, we initially evaluated the antifungal activities at concentrations ranging from 250 μg/mL to 1000 μg/mL, and the commercial fungicide Triadimefon was selected as the positive control. Triadimefon, a systemic fungicide in the triazole family of chemicals, is highly effective against various leaf spot diseases in many different crops. It has protective, curative, and eradicative effects, and has been widely used worldwide. The antifungal results are shown in Table 2. In general, all the derivatives were able to inhibit the growth of all three plant-pathogenic fungi. Furthermore, the chitosan derivatives displayed a high dose-dependent effect. It can be seen that the antifungal activity of ATUCS and CATUCS was significantly enhanced compared with original chitosan, especially at high concentrations. The antifungal activity of ATUCS against *G. theae-sinensis* and *S. solani* can even pass 90% at 1000 μg/mL, which is better than that of the positive control, Triadimefon. In addition, ATUCS exhibited better antifungal properties than CATUCS. It was inferred that various substituents in thiourea chitosan had distinct impacts on the antifungal activity. These results were also in accordance with earlier studies [28,29,30].

**Figure 6:**
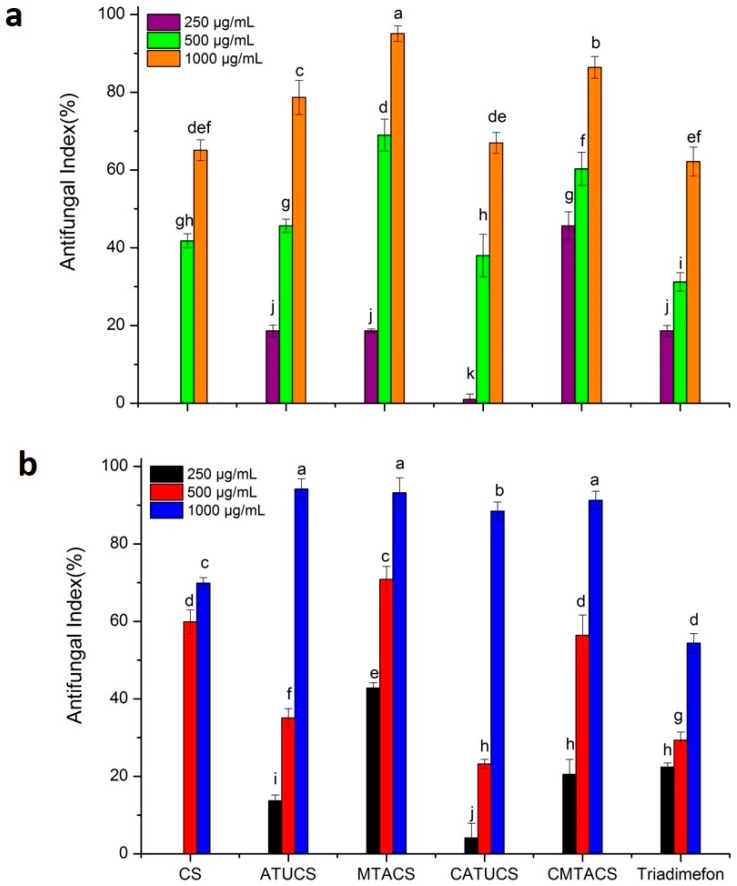
Inhibitory effect of chitosan (CS), acetyl thiourea chitosan (ATUCS), 3-methyl-1,2,4-triazolyl chitosan (MTACS), chloracetyl thiourea chitosan (CATUCS) and 3-chloromethyl-1,2,4-triazolyl chitosan (CMTACS) derivative on *A. porri* (**a**) and *G. theae-sinensis* (**b**).

It was observed that MTACS and CMTACS exhibited a much stronger inhibitory effect on the selected crop pathogenic fungi than the original chitosan, with the inhibitory index of the derivatives ranging from 18.6% to 100%. Compared with ATUCS and CATUCS, MTACS and CMTACS displayed significantly enhanced antifungal activity in general. For example, the antifungal index of MTACS and CMTACS was (95.1 ± 2.0) and (86.4 ± 2.8), while that of ATUCS and CATUCS was (78.7 ± 4.4) and (67.0 ± 2.7), respectively (Figure 6a,b). In particular, MTACS, for which the antifungal index against *A. porri* and *G. theae-sinensis* was more than 60% at 500 μg/mL, displayed even better antifungal activity than that of the positive control, Triadimefon (about 30%). Thereby, it was concluded that the cyclization of acyl thiourea chitosan (TUCS) was an efficient method to enhance its antifungal activity. The combination of triazolyl groups with chitosan increased both the antifungal activity and solubility of CS. It is known that *G. theae-sinensis*, one of the most destructive pathogens of tea, can infect the tender leaves of the tea and decrease the yield and quality. *A. porri*, which can cause onion purple blotch disease, causes serious onion losses every year. Therefore, there may be potential applications for the prevention and control of diseases in tea plants and onions due to the scarcity of green fungicides available.

The antifungal activity of the derivatives against *S. solani* is shown in Table 2. The inhibitory index of the derivatives ranged from 26.8% to 100%. Compared with CS, ATUCS, CATUCS, MTACS, and CMTACS exhibited significantly better antifungal activity. This means that introduction of the substituent group at the C-2 position increased the antifungal activity of CS. However, the antifungal activity of the chitosan derivatives against *S. solani* was not comparable to that of Triadimefon. At a low concentration of 250 μg/mL, Triadimefon inhibited *S. solani* by 100%. Hence, it was deduced that the fungus was much more sensitive to Triadimefon.

Based on the above antifungal evaluation results, it was concluded that structural optimization of TUCS via ring closure reaction is a feasible method to improve its activity. It was proven that the TACS exhibited more potent activity against the selected fungi in contrast with chitosan and TUCS. Both MTACS and CMTACS had broad-spectrum antifungal activity. In conclusion, the results of the antifungal tests indicated differences in the sensitivity of the three fungi against the sample tested, with *A. porri* and *G. theae-sinensis* being the most inhibited.

## 3. Materials and Methods

### 3.1. Materials

Chitosan was provided by Qingdao Baicheng Biochemical Corp (Qingdao, China). Its degree of deacetylation is 87% and average molecular weight is 230 KDa. Acetyl chloride, chloroacetyl chloride, ammonium thiocyanate, hydrazine hydrated, acetic anhydride, ethanol, and the other chemical reagents and solvents were all analytical grade purchased from Sinopharm Chemical Reagent Co., Ltd. (Shanghai, China) and Sigma-Aldrich (Saint Louis, MO, USA). Three crop-threatening pathogenic fungi, *Stemphylium solani* weber (*S. solani*), *Alternaria porri* (*A. porri*), and *Gloeosporium theae-sinensis* (*G. theae-sinensis*), were supplied by the Qingdao Academy of Agricultural Sciences.

### 3.2. Analytical Methods

Fourier transform infrared (FTIR) spectra were recorded in the 4000–400 cm^−1^ region using a Thermo Fisher Scientific (Waltham, MA, USA) Nicolet iS10 FT-IR spectrometer in KBr discs. 1H-NMR spectrum was performed by nuclear magnetic resonance (NMR) with a JEOL (Akishima, Japan) JNM-ECP600 spectrometer, using CD3COOD and D2O as solvents. The elemental analysis (C, H, N, and S) was performed on a Vario EL-III elemental analyzer (Elementar, Hesse, Germany). X-ray diffraction (XRD) measurement of the powder samples were investigated with a D8 Advance diffractometer (Bruker, Billerica, MA, USA) with Cu target (λ = 0.154 nm) at 40 Kv and the scanning scope of 2θ was 5–40°. The surface morphology of the chitosan derivatives was analyzed with scanning electron microscopy (SEM) by using KYKY-2800B SEM. Differential scanning calorimetry (DSC) was performed using The Pyris Diamond DSC (PerkinElmer, Waltham, MA, USA). The samples were heated from 50 °C to 300 °C. Molecular geometry optimizations for the chitosan derivatives were carried out using the Gaussian 09 program.

### 3.3. Synthesis of 3-Methyl-1,2,4-triazolyl Chitosan (MTACS) Derivative and 3-Chloromethyl-1,2,4-triazolyl Chitosan (CMTACS) Derivative

The preparation of acyl thiourea chitosan (TUCS) was according to t [22]. Firstly, chitosan (6 g, 37.3 mmol) was immersed into (DMF:HAc = 1:1) solvent. After 10 h, 40 mL dichloromethane solution containing 40 mmol acyl isothiocyanate was added slowly at room temperature. Then the reacted mixture was heated to 90 °C and stirred for 3 h. Next, the reactant was filtered and washed with ethanol and dried at 50 °C. The solid product was put in a Soxhlet extractor with anhydrous ethanol continuous extraction for 6 h. After that, acetyl thiourea chitosan (ATUCS, yield: 65%) and chloroacetyl thiourea chitosan (CATUCS, yield: 57%) were obtained via drying; ATUCS: DS: 0.337; ^1^H-NMR: 1.82 ppm (-COCH3), 2.93–3.72 ppm (pyranose rings (H2, H5, H6, H3, H4)), 7.70 ppm (NHCS); FT-IR (thin film): v 3400 (NH_2_ and OH), v 1726 (C=O), v 1256 (C=S); Elemental data: (C: 0.3966, N: 0.0867, H: 0.0673, S: 0.0411); CATUCS: DS: 0.27; ^1^H-NMR: 1.82 ppm (-COCH3), 2.93–3.72 ppm (pyranose rings (H2, H5, H6, H3, H4), 8.02 ppm (NHCS); FT-IR (thin film): v 3400 (NH_2_ and OH), v 1733 (C=O), v 1249 (C=S); Elemental data: (C: 0.3830, N: 0.0781, H: 0.0680, S: 0.0301).

A mixture of chitosan acyl thiourea (10 mmol), anhydrous ethanol (40 mL), and acetic anhydride (0.1 mL) was heated to 60 °C. Then the ethanol solution (40 mL) containing 85% hydrazine (2.0 mL) was drop-wise added, the reaction mixture refluxed for 10 h. Workup is same as the above method, 3-methyl-1,2,4-triazolyl chitosan (MTACS, yield 62% ) and 3-chloromethyl-1,2,4-triazolyl chitosan (CMTACS, yield 59%) was obtained; MTACS: DS: 0.244; ^1^H-NMR: 1.82 ppm (-COCH3), 2.93–3.72 ppm (pyranose rings (H2, H5, H6, H3, H4)),10.65 ppm (triazolyl-H); FT-IR (thin film):v 3400 (NH_2_ and OH), v 1640 (C=N); Elemental data: (C: 0.3739, N:0.0753, H: 0.0618, S: 0.0088); CMTACS: DS: 0.19; ^1^H-NMR: 1.82 ppm (-COCH3), 2.93–3.72 ppm (pyranose rings (H2, H5, H6, H3, H4), 10.67ppm (triazolyl-H); FT-IR (thin film):v 3400 (NH_2_ and OH), v 1643 (C=N); Elemental data: (C: 0.3677, N:0.1035, H: 0.0635, S: 0.0093).

### 3.4. Antifungal Activity in Vitro

Antifungal assays were tested in vitro by a mycelium growth rate test. Chitosan and its derivatives were dissolved in 1% (*v*/*v*) acetic acid at an original concentration of 2% (*w*/*v*). The solutions were autoclaved at 121 °C for 20 min and mixed with sterile molten potato dextrose agar (PDA) to obtain final concentrations of 250 μg/mL, 500 μg/mL, and 1000 μg/mL, respectively. When the agar was cooled, the mycelium of fungi was transferred to the plates and then incubated at 27 °C. The mixed medium without sample was used as the blank control; Triadimefon was treated as the positive control. When the mycelium of fungi reached the edges of the blank control plate, the antifungal index was calculated with the following equation:

$$\mathrm{Antifungal}\;\mathrm{index}=\left(1-\frac{D_{t}}{D_{b}}\right)\times 100$$

Here, D_t_ is the colony diameter in the test plate and D_b_ is the colony diameter in the blank control.

Three replicates of each test were carried out and the results were averaged. Data were presented as mean ± standard error. Differences among different treatments were analyzed using SPSS version 19.0 (SPSS, Inc., Chicago, IL, USA). One-way analysis of variance (ANOVA) and Duncan’s multiple range tests were used to identify the significance of the differences (*p* < 0.05) between the means. The graphs were generated with Origin 8.5.

## 4. Conclusions

In this study, 3-methyl-1,2,4-triazolyl chitosan (MTACS) and 3-chloromethyl-1,2,4-triazolyl chitosan (CMTACS) were successfully prepared via cyclization of acyl thiourea chitosan (TUCS) and their structures were well confirmed by FT-IR, ^1^H-NMR, elemental analysis, DSC, XRD, and SEM. Their inhibitory effects on *S. solani*, *A. porri*, and *G. theae-sinensis* were investigated. It was shown that MTACS and CMTACS exhibited more broad-spectrum and higher antifungal properties compared with chitosan, ATUCS, and CATUCS. Moreover, they displayed even better antifungal activity against *A. porri* and *G. theae-sinensis* than the positive control, Triadimefon. Hence, it was inferred that structural optimization of TUCS via ring closure reaction is a feasible method to improve its activity. All the data described here strongly suggest that TACS are promising antifungal candidates that can be used as lead structures for new fungicide development.

## Figures and Tables

**Table 1:** Elemental analysis results and degree of substitution of chitosan (CS), acetyl thiourea chitosan (ATUCS), 3-methyl-1,2,4-triazolyl chitosan (MTACS), chloracetyl thiourea chitosan (CATUCS), and 3-chloromethyl-1,2,4-triazolyl chitosan (CMTACS) derivative.

Compounds	Elemental Analysis (%)				DS_thiourea_ (%)	DS_triazole_ (%)
	C	N	H	S		
CS	40.05	7.29	6.41	–	–	–
ATUCS	39.66	8.67	6.73	4.11	33.7	–
CATUCS	38.30	7.81	6.80	3.01	27.0	–
MTACS	37.39	7.53	6.18	0.88	9.3	24.4
CMTACS	36.77	10.35	6.35	0.93	8.0	19.0

**Table 2:** Antifungal activity of chitosan (CS), acetyl thiourea chitosan (ATUCS), 3-methyl-1,2,4-triazolyl chitosan (MTACS), chloracetyl thiourea chitosan (CATUCS), and 3-chloromethyl-1,2,4-triazolyl chitosan (CMTACS) derivative against *A. porri*, *G. theae-sinensis*, and *S. solani*.

Sample	Concentration (μg/mL)	*A. porri*	*G. theae-sinensis*	*S. solani*
		Antifungal Index *	Antifungal Index *	Antifungal Index *
CS	250	0 ^k^	0 ^j^	12.3 ± 1.3 ^i^
	500	41.8 ± 1.8 ^g,h^	59.9 ±3.1 ^d^	41.2 ± 2.9 ^g^
	1000	65.1 ± 2.7 ^d,e,f^	69.9 ± 1.4 ^c^	50.9 ± 3.6 ^f^
ATUCS	250	18.6 ± 1.5 ^j^	13.7 ± 1.5 ^i^	27.7 ± 1.2 ^h^
	500	45.7 ± 1.7 ^g^	35.1 ± 2.4 ^f^	54.7 ± 2.7 ^e,f^
	1000	78.7 ± 4.4 ^c^	94.2 ± 2.6 ^a^	90.3 ± 3.0 ^b^
MTACS	250	18.6 ± 0.5 ^j^	42.8 ± 1.4 ^e^	60.5 ± 2.5 ^e^
	500	69.0 ± 4.1 ^d^	70.9 ± 3.3 ^c^	82.6 ± 4.2 ^c^
	1000	95.1± 2.0 ^a^	93.2 ± 3.9 ^a^	95.2 ± 3.7 ^a,b^
CATUCS	250	1.0 ± 1.4 ^k^	4.1 ± 3.8 ^j^	26.8 ± 2.6 ^h^
	500	38.0 ± 5.5 ^h^	23.2 ± 1.2 ^h^	54.7 ± 1.4 ^e,f^
	1000	67.0 ± 2.7 ^d,e^	88.5 ± 2.3 ^b^	70.1 ± 2.2 ^d^
CMTACS	250	45.7 ± 3.6 ^g^	20.5 ± 3.9 ^h^	42.2 ± 6.3 ^g^
	500	60.3 ± 4.3 ^f^	56.4 ± 5.3 ^d^	71.1 ± 2.4 ^d^
	1000	86.4 ± 2.8 ^b^	91.3 ± 2.3 ^a^	100 ^a^
Triadimefon	250	18.6 ± 1.4 ^j^	22.4 ± 1.1 ^h^	100 ^a^
	500	31.2 ± 2.4 ^i^	29.3 ± 2.1 ^g^	100 ^a^
	1000	62.2 ± 3.7 ^e,f^	54.4 ± 2.5 ^d^	100 ^a^

* Values are means ± standard error; data in columns with different letters are statistically different according to Duncan’s multiple range tests (*p* = 0.05); each treatment consisted of three replicates.

## Abbreviations

The abbreviation in this article was shown as follows:

3-methyl-1,2,4-triazolyl chitosan	MTACS
3-chloromethyl-1,2,4-triazolyl chitosan	CMTACS
Acetyl thiourea chitosan	ATUCS
Chloroacetyl thiourea chitosan	CATUCS
Chitosan	CS
*Stemphylium solani weber*	*S. solani*
*Alternaria porri*	*A. porri*
*Gloeosporium theae-sinensis*	*G. theae-sinensis*
Fourier transform infrared	FTIR
Nuclear magnetic resonance	NMR
X-ray diffraction	XRD
Differential scanning calorimetry	DSC
Scanning electron microscopy	SEM
Potato dextrose agar	PDA
N,N-Dimethylformamide	DMF
